# HPV Genotype, AGC Categories, and Age-Stratified Immediate Prevalence of Precancers and Cancers in Women with Atypical Glandular Cells with or without Concurrent Squamous Abnormal Cytology

**DOI:** 10.7150/jca.105805

**Published:** 2025-03-31

**Authors:** Xin Zhou, Zicheng Huang, Wanrun Lin, Suming Huang, Huijuan Zhang, Wenxin Zheng, Yudong Wang, Feng Zhou

**Affiliations:** 1 Department of Pathology, The International Peace Maternal and Child Health Hospital, School of Medicine, Shanghai Jiao Tong University, Shanghai, China, 200030.; 2 Shanghai Key Laboratory of Embryo Original Diseases, Shanghai, China, 200030.; 3 Department of Oncology, The International Peace Maternal and Child Health Hospital, School of Medicine, Shanghai Jiao Tong University, Shanghai, China, 200030.; 4 Laboratory of Pathology, National Cancer Institute, National Institutes of Health, Bethesda, MD, USA, 20892.; 5 Department of Pathology, Department of Obstetrics and Gynecology, University of Texas Southwestern Medical Center, Dallas, TX, USA, 75390.; 6 Harold C. Simon Comprehensive Cancer Center, University of Texas Southwestern Medical Center, Dallas, TX, USA, 75390.

**Keywords:** Papanicolaou (Pap) test, Atypical glandular cells, coexisting glandular and squamous cells abnormalities, histological follow-up, risk stratification

## Abstract

**Objectives:** Limited data exists on Papanicolaou (Pap) tests involving atypical glandular cells (AGC) with or without concurrent squamous cell abnormalities (Sq), hindering the reproducibility of results. This study aims to stratify the risk of precancers and cancers based on distinct high-risk human papillomavirus (hrHPV) genotyping, AGC categories, and age groups among women with AGC with or without concurrent squamous cell abnormalities.

**Methods:** This retrospective analysis examined Pap smear patient data from January 2019 to December 2023, including 54 AGC + Sq cases and 974 cases with AGC-Alone. Among these, 799 patients (including 43 AGC + Sq cases and 756 AGC-Alone cases) had HPV testing results, and 769 (including 43 AGC + Sq cases and 726 AGC-Alone cases) had subsequent histological follow-up data.

**Results:** In the total cohort, 5.25% (54 cases) were AGC + Sq, and 94.75% (974 cases) were AGC-Alone. The detection rates of high-grade glandular lesions (AIS+/AEH+) and adenocarcinoma (AC) were significantly higher in AGC patients over 65 years compared to other age groups (p = 0.000444 and p < 0.0001, respectively), while no significant differences were observed for high-grade squamous lesions (HSIL+) (p = 0.791) or squamous carcinoma (SCC) (p = 0.909). The prevalence of AIS+/AEH+ was significantly higher in HPV-16 (28.6%) and HPV-18 (50.0%) positive groups compared to the HPV-negative (10.4%) and other hrHPV types positive groups (6.3%) (p < 0.0001). Notably, the AGC + Sq group exhibited a higher prevalence of isolated squamous lesions, as well as glandular lesions with concurrent squamous involvement, compared to the AGC-Alone group (p = 0.001). Additionally, increased AC risk was observed in older AGC + Sq women at the 50-year cutoff, although no significant association was found between HPV genotype and immediate histology in the AGC + Sq group.

**Conclusions:** A comprehensive approach that incorporates cytological results, hrHPV status, and age offers more effective stratification of AGC patients, leading to more precise management. While hrHPV testing and age provide valuable insights, relying solely on hrHPV results for triaging AGC + Sq cases is inadequate.

## Introduction

Despite significant declines in the incidence and mortality rates of cervical squamous cell carcinoma (SCC) due to advancements in screening and vaccination, the incidence of cervical adenocarcinoma is increasing 1, 2. This shifting trend highlights that while progress has been made in controlling SCC, the epidemiological landscape of cervical adenocarcinoma is evolving, thereby presenting new challenges to existing screening strategies. Consequently, the accurate diagnosis of AGC for the early detection of glandular tumors in the female reproductive tract remains a significant challenge in gynecological cytopathology 3. Typically, Papanicolaou (Pap) tests report either isolated squamous cell abnormalities or glandular cell abnormalities. However, the concurrent presence of glandular and squamous cell abnormalities is not common. Such co-occurrence may suggest either squamous lesions involving the endocervical glands or, less frequently, simultaneous squamous and glandular lesions 4, 5. This scenario may result in clinicians lacking sufficient experience and guidance for managing these cases.

Managing AGC cases presents multifaceted challenges due to the low prevalence of AGC (<1.0%) and the significant interobserver variability, which is due to the lack of standardized cytological criteria 6-8. Furthermore, the correlation between cytological and histological findings is often inadequate. This discrepancy shows a broad range of benign and malignant conditions, with a high proportion of benign findings in the assessments. Moreover, a considerable number of AGC cases also reveal potential high-grade squamous lesions, adenocarcinoma in situ (AIS) of the cervix, and invasive cancers of the cervix, endometrium, ovaries, fallopian tubes, or other extragenital sites 4, 9.

Given the inter-observer variability in clinical evaluation of AGC cytology results, researchers have sought to stratify the risk of precancerous and cancerous lesions in AGC cases based on patient age and hrHPV testing results to optimize management and avoid unnecessary interventions 10-13. However, studies addressing the histological outcomes in cases with both squamous and glandular cells abnormalities are still limited 14. Therefore, the main aim of this study was to investigate the prevalence and the distribution of hrHPV among women with AGC with or without Sq, to evaluate the immediate risk of precancers and cancers in women with AGC with or without Sq according to hrHPV genotypes, AGC categories, and age in Chinese population. Thereby shedding light on the utility of HPV genotyping, AGC categories, and age stratification in the management of these patients.

## Methods and Materials

### Sample Collection

With approval from The Institutional Review Board and Ethics Committee of International Peace Maternity and Child Health Hospital Affiliated to Shanghai Jiao Tong University School of Medicine, we conducted a retrospective review of patients diagnosed with glandular cells abnormalities who underwent cervical Pap testing from January 2019 to December 2023. Inclusion Criteria: Females with a history of sexual activity who were non-pregnant; all subjects with a clear diagnosis of cervical biopsy pathology; subjects underwent Liquid-Based Cytology Test (LCT) testing, with results indicating AGC; subjects with no autoimmune disorders. Exclusion Criteria: subjects with unreliable follow-up; subjects with history of hysterectomy or malignant tumors; subjects with history of other malignancies or pelvic radiotherapy/chemotherapy. We retrieved data from medical records, including patient age at the time of AGC diagnosis, available HPV test results, and follow-up histopathological outcomes. Histopathological samples were collected through various procedures, including colposcopic endocervical curettage, cervical biopsy, endometrial curettage, loop electrosurgical excision procedure (LEEP), or cone biopsy, as well as hysterectomy. Fourteen hrHPV genotypes (16, 18, 31, 33, 35, 39, 45, 51, 52, 56, 58, 59, 66, and 68) were tested. Positive hrHPV samples were categorized into four types: HPV16, HPV18, HPV16/18 dual-positive, and other hrHPV types.

### Follow-Up Histopathological Diagnoses

Histopathological results were classified into four general categories: (1) Negative/ Benign, indicating no pathological changes or benign/reactive alterations; (2) Low-grade squamous intraepithelial lesion (LSIL); (3) High-grade squamous lesion (HSIL+), including either HSIL or squamous cell carcinoma (SCC); and (4) High-grade glandular lesions (AIS+/AEH+), encompassing cervical adenocarcinoma in situ (AIS), atypical endometrial hyperplasia (AEH), or adenocarcinoma (AC). In cases of diagnostic ambiguity, multiple pathologists reviewed the cases to reach a consensus. Precancerous lesions were defined as HSIL, AIS, and AEH.

### Statistical Analysis

**Figure 1:**
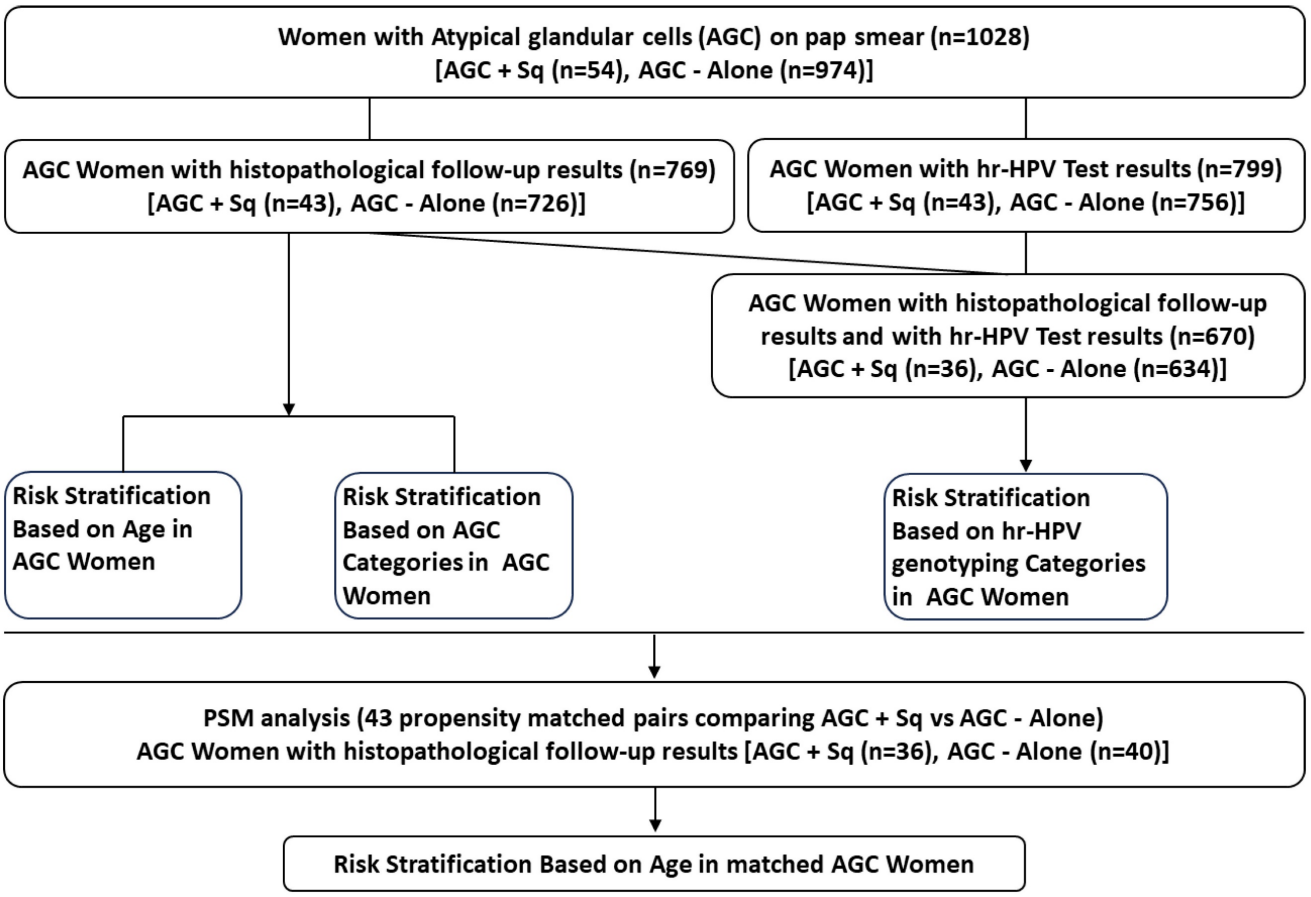
Schematic Overview of Data Analysis Workflow.

Categorical variables were analyzed using Pearson's chi-square test, Fisher's exact test, and Kruskal-Wallis test. Continuous variables with a normal distribution were assessed with the t-test, while those with a non-normal distribution were evaluated using the Mann-Whitney U test. Propensity score matching (PSM) was employed to minimize selection bias by matching HPV types and AGC categories. A 1:1 nearest neighbor matching approach was applied using a caliper width of 0.05 without replacement, based on each patient's estimated propensity score. Following matching, 43 patients were selected for each group. All statistical analyses were performed using SPSS software (version 25.0, IBM) and R software (version 4.4), with *p*-values < 0.05 considered statistically significant. The data analysis workflow is illustrated in Figure 1.

## Results

### Demographic Characteristics of Patients

**Figure 2:**
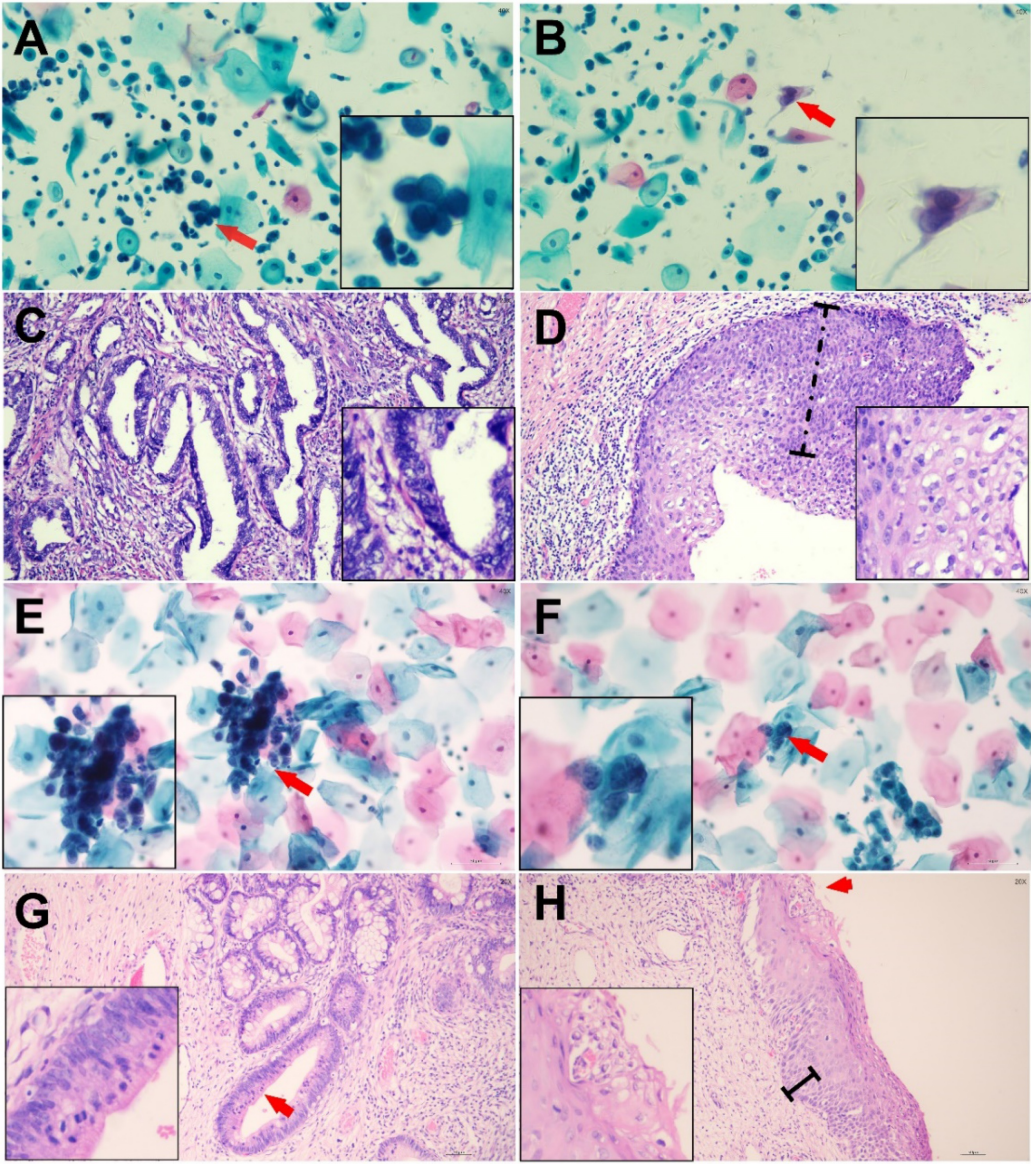
Representative Cytologic and Histologic Findings in Two Cases of Abnormal Glandular Cells with Concurrent Squamous Abnormalities Confirmed by Histologic Follow-up. Case 1: Endocervical Adenocarcinoma (ECA) with High-Grade Squamous Intraepithelial Lesion (HSIL) (Cytologic diagnosis: AGC-FN/ASC-H) (Panels A-D): (A) AGC-FN, arrow indicates a tightly packed cluster of glandular cells with a high nuclear-to-cytoplasmic ratio, enlarged nuclei, and coarse chromatin. (B) HSIL, arrow highlights abnormal squamous cells with eosinophilic cytoplasm, hyperchromatic, enlarged nuclei, and irregular nuclear contours. (C) Endocervical adenocarcinoma, disorganized glands of varying shapes and sizes composed of columnar tumor cells. (D) HSIL, dysplastic changes involving more than two-thirds of the epithelial thickness, with loss of nuclear polarity and numerous koilocytic cells on the surface. Case 2: Endocervical Adenocarcinoma In Situ (AIS) with Low-Grade Squamous Intraepithelial Lesion (LSIL) (Cytologic diagnosis: AGC-FN/ASC-US) (Panels E-H): (E) AGC-FN, arrow indicates atypical glandular cells with a high nuclear-to-cytoplasmic ratio. (F) ASC-US, arrow points to squamous cells with enlarged nuclei. (G) AIS, abnormal endocervical glands adjacent to normal glands without stromal reaction. The neoplastic cells form stratified, crowded clusters with hyperchromatic nuclei and atypical mitoses at the luminal borders (arrow). (H) LSIL, mild nuclear polarity disorder involving the basal one-third of the epithelium, with variable nuclear sizes, mitotic figures, and surface koilocytic cells (arrow).

**Figure 3:**
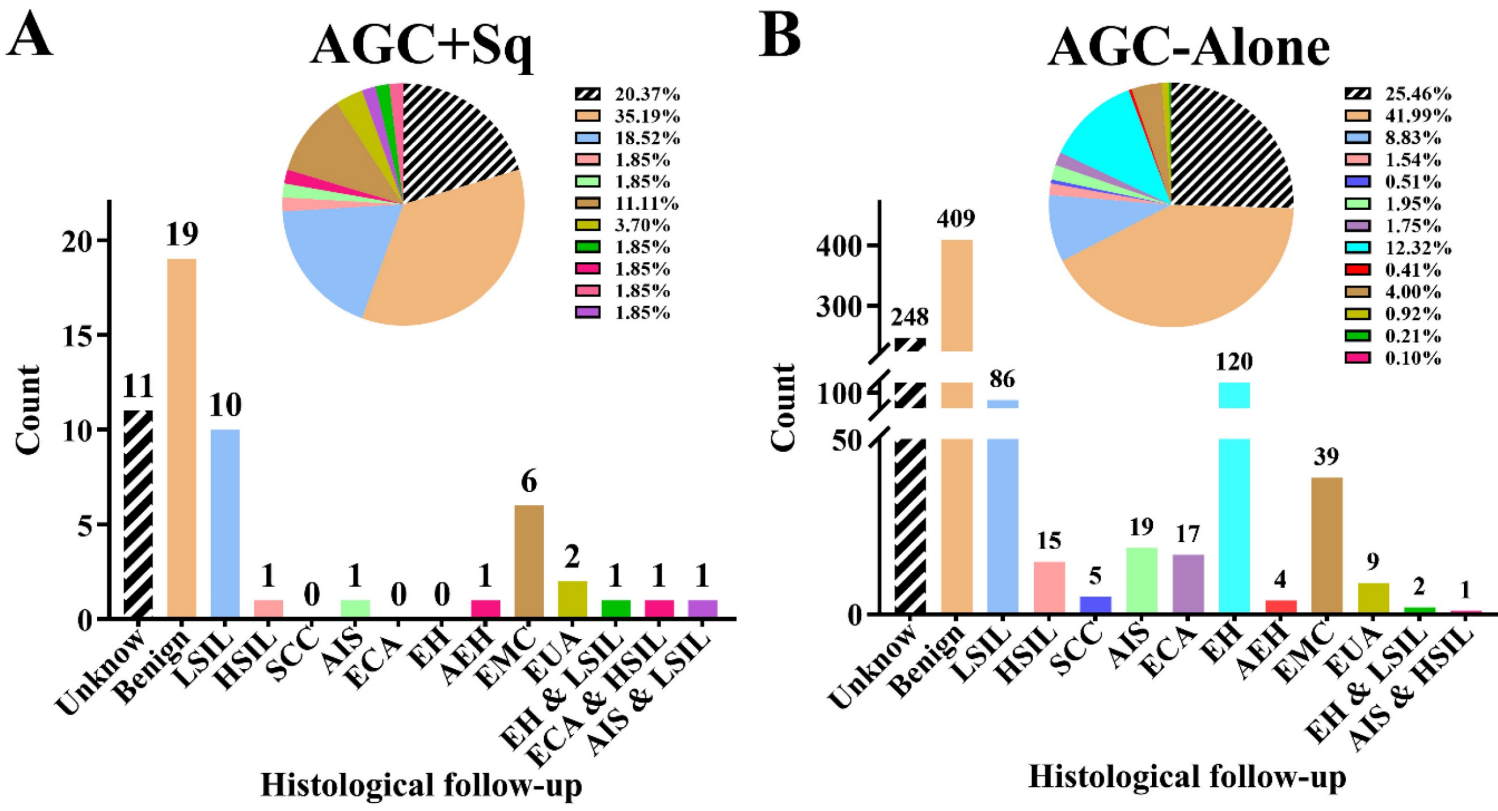
Histologic outcomes in women with atypical glandular cells (AGC) with or without concurrent squamous cell abnormalities. AGC + Sq group (A), AGC-alone group (B). **Abbreviations:** AC, adenocarcinoma; AEH: atypical endometrial hyperplasia; AGC, atypical glandular cell; AGC + Sq, atypical glandular cells with concurrent squamous cell abnormalities; AGC-alone, atypical glandular cells without concurrent squamous cell abnormalities; AIS, adenocarcinoma in situ; EH, endometrium hyperplasia without atypia; ECA, endocervical adenocarcinoma; EMC, endometrial carcinoma; EUA, Extrauterine adenocarcinoma; HSIL, high-grade squamous intraepithelial lesion; SCC, squamous cell carcinoma; LSIL, low-grade squamous intraepithelial lesion.

This study included a total of 1,028 cases with atypical glandular cells (AGC) on Pap tests. The cohort was divided into two groups: 54 cases (5.25%) of AGC with concurrent squamous abnormalities (AGC + Sq) and 974 cases (94.75%) of AGC alone (AGC-Alone). The associated squamous cells abnormalities in the AGC + Sq group comprised 40 cases of atypical squamous cells of undetermined significance (ASC-US), 3 cases of LSIL, 7 cases of atypical squamous cells that- cannot exclude high-grade squamous intraepithelial lesion (ASC-H), and 4 cases of HSIL. As detailed in Table 1, the mean age of patients in the AGC + Sq group was 48.22 ± 12.79 years, compared to 45.25 ± 10.87 years in the AGC-Alone group, with the AGC + Sq group being significantly older (*p* = 0.032). Additionally, the proportion of older patients in the AGC + Sq group increased with age compared to the AGC-Alone group (p < 0.0001). In the AGC-Alone group, the majority were categorized as AGC-NOS (368/974 [37.8%]), whereas AGC + Sq cases were predominantly AGC-FN (22/54 [40.7%]) (*p* < 0.0001). hrHPV testing results were available for 799 cases (77.7%), with a positivity rate of 17.9% (143/799). The AGC + Sq group had a slightly higher hrHPV positivity rate (27.9%) compared to the AGC-Alone group (17.3%) (*p* = 0.078). Histologic follow-up was obtained for 769 cases (74.8%). The representative cytological and follow-up histologic images were showed in Figure 2 and the correlation results are shown in Figure 3. Among the histologic follow-up, only 6 patients exhibited both squamous and glandular lesions: 3 with LSIL and endometrial hyperplasia (EH), 1 with HSIL and AIS, 1 with LSIL and AIS, and 1 with HSIL and cervical adenocarcinoma (ECA). The detection rates of precancerous lesions and cancers in AGC patients were 30.4% (43/1,028) and 9.8% (79/1,028), respectively, with AGC + Sq cases showing significantly higher immediate pathological severity compared to AGC-Alone cases (*p* < 0.0001).

### Specific HPV Genotypes and their Immediate Histological Correlation Results among AGC Women

Among AGC women with available HPV testing results, 82.1% (656/799) were HPV-negative, 4.0% (32/799) were HPV-16 positive, 2.8% (22/799) were HPV-18 positive, 0.3% (2/799) were positive for both HPV-16 and HPV-18, and 10.9% (87/799) were positive for other hrHPV types. HPV genotype distribution among AGC + Sq and AGC-Alone women is shown in Table 1.

A total of 670 cases (including 36 AGC + Sq cases and 634 AGC-Alone cases) had concurrent data on HPV genotypes and immediate histological results. Among these 670 AGC women, 3.4% (23/670) were diagnosed with HSIL+, and 11.9% (80/670) with AIS+/AEH+. After stratifying by specific hrHPV genotypes, we observed that the prevalence of HSIL+ was 32.1% in the HPV-16 positive group and 20.0% in the HPV-18 positive group, compared to 7.5% and 0.7% in the other hrHPV positive group and HPV-negative group, respectively (*p* < 0.0001). Despite lower overall rates of SCC in the HPV-16 (7.1%) and HPV-18 (10.0%) positive groups, these were significantly higher than in the other hrHPV positive (1.3%) and HPV-negative groups (0%) (*p* < 0.0001). Similarly, the prevalence of AIS+/AEH+ in the HPV-16 (28.6%) and HPV-18 (50.0%) positive groups was significantly higher compared to the HPV-negative (10.4%) and other hrHPV types positive groups (6.3%) (*p* < 0.0001). In the HPV-18 and HPV-16 positive groups, adenocarcinoma (AC) prevalence was 15% and 17.9%, respectively, compared to 5.0% and 7.8% in the other high-risk HPV positive and HPV-negative groups (*p* = 0.0089). Due to the limited number of cases (n = 2), HPV-16 and HPV-18 dual positive women was not conducted in the statistical analysis.

The cytologic subgroup analysis revealed that in AGC-Alone women, the risk stratification of high-grade squamous and glandular lesions according to HPV genotype was similar to that observed in the broader AGC cohort. The HPV-16 and HPV-18 positive groups had higher detection rates of high-grade squamous and glandular lesions compared to other groups (*p* < 0.0001). However, no significant association was found between HPV genotype and immediate histology in the AGC + Sq group. Detailed results are presented in Table 2.

### Immediate Histological Correlation Results among AGC Women according to AGC Categories

As shown in Table 3, when stratifying AGC women by the AGC categories, the AGC-FN group showed a high prevalence of HSIL+ (7.3%), SCC (3.1%), AIS+/AEH+ (51.0%), and AC (40.6%), all significantly higher than in other groups (*p* = 0.001, *p* = 0.004, *p* < 0.0001, *p* < 0.0001). In the AGC-Alone group, the prevalence of HSIL+, SCC, AIS+/AEH+, and AC in the AGC-FN group was also significantly higher than in other groups (*p* = 0.002, p = 0.002, *p* < 0.0001, *p* < 0.0001). Moreover, in the AGC + Sq group, the prevalence of AIS+/AEH+ was significantly higher in the AGC-FN (47.1%) and AEM (37.5%) subgroups compared to other groups (*p* = 0.011). Similarly, AC rates were significantly higher in the AGC-FN (35.3%) and AEM (37.5%) groups compared to other groups (*p* = 0.012).

### Age-Stratified Immediate Histological Correlation Results among AGC Women

Analysis of HPV genotype distribution by age among AGC women revealed that of the 769 cases with follow-up results, 0.5% (4/769) were under 25 years old, with 25% (1/4) testing positive for other hrHPV types. Among those aged 25-39 years (28.3%, 218/769), 4.1% (9/218) were HPV-16 positive, 4.6% (10/218) were HPV-18 positive, 0.5% (1/218) were positive for HPV-16/HPV-18/45, and 11.5% (25/218) were positive for other hrHPV types. In the 40-65 age group (65.3%, 502/769), 3.4% (17/502) were HPV-16 positive, 1.8% (9/502) were HPV-18 positive, 0.2% (1/502) were positive for HPV-16/HPV-18, and 9.4% (47/502) were positive for other hrHPV types. Among those over 65 years (5.9%, 45/769), 4.4% (2/45) were HPV-16 positive, 2.2% (1/45) were HPV-18 positive, and 8.9% (4/45) were positive for other hrHPV types.

The prevalence of AIS+/AEH+ varied by age: 25% (1/4) in those under 25 years, 10.6% (23/218) in the 25-39 age group, 12.4% (62/502) in the 40-65 age group, and 33.3% (15/45) in those over 65 years. Similarly, AC rates were 25% (1/4) in those under 25 years, 4.1% (9/218) in the 25-39 age group, 10.0% (50/502) in the 40-65 age group, and 31.1% (14/45) in those over 65 years. The detection rates of AIS+/AEH+ and AC in AGC women over 65 years were significantly higher compared to other age groups (*p* = 0.000444 and p < 0.0001, respectively), while no significant differences were observed for HSIL+ (*p* = 0.791) and SCC (*p* = 0.909). Subgroup analysis indicated that in AGC-Alone women, the prevalence of AIS+/AEH+ and AC in those over 65 years was higher than in other age groups (*p* = 0.002 and *p* < 0.0001, respectively). Detailed results are presented in Table 4.

A detailed age-specific analysis revealed a significant increase in high-grade glandular lesion prevalence from age 45 onward when using 40 years as the initial threshold with five-year age intervals (p < 0.0001). Notably, the risk of adenocarcinoma exhibited a marked rise at age 40 (p = 0.002). In contrast, no significant differences were observed in HSIL+ or SCC across various age cutoffs. Subgroup analysis demonstrated comparable risk stratification patterns between AGC-Alone and AGC + Sq groups across age thresholds, with AC risk increasing in AGC + Sq women aged 50 and older (p = 0.019) (Table 5).

To mitigate potential biases related to HPV type and AGC category, PSM was applied, and the risk of precancer and cancer was reassessed between older and younger age groups. As shown in Table S1, among AGC women aged >65, the detection rates of AIS+/AEH+ and AC were no longer significantly different from those in younger age groups. However, in the AGC-Alone group, women aged 40-65 exhibited a significantly higher prevalence of HSIL+ and SCC (p = 0.011 and p = 0.048, respectively). Age stratification revealed that the risk pattern in the AGC-Alone group remained largely unchanged after matching, whereas in the AGC + Sq group, a trend toward increased AC risk was observed in older women when using a 55-year cutoff (p = 0.063) (Table S2).

## Discussion

Although the simultaneous occurrence of squamous and glandular lesions in histology is not common, the co-existence of squamous and glandular cells abnormalities in Pap tests has garnered considerable attention 15, 16,. To date, literature concerning the dual interpretation of squamous and glandular cells abnormalities using Bethesda terminology and their subsequent histopathological relevance remains sparse. Our findings align with previous studies but also introduce several novel observations.

Previous studies have indicated that the mean age of the AGC + Sq cohort was notably lower than that of the AGC-Alone group 17, 18. However, our findings suggest a different trend, with a markedly higher proportion of older women (over 65 years) in the AGC + Sq group (16.7%) compared to the AGC-Alone group (6.1%). This finding contrasts with that of earlier research and may be attributed to demographic differences within our study population or evolving patterns in HPV epidemiology. The higher age distribution in the AGC + Sq group underscores the importance of targeted screening and vigilant follow-up in older women presenting with concurrent squamous abnormalities. Additionally, the AGC + Sq group predominantly comprised AGC-FN (40.7%), whereas the AGC-Alone group was primarily AGC-NOS (37.8%). This distribution differs from that reported by Lin et al. 17, who identified AGC-NOS as the most common in the AGC-Alone group and AEC as predominant in the AGC + Sq cases. These discrepancies may be attributed to variations in diagnostic criteria or population characteristics.

Regarding hrHPV infection, multiple studies have shown a significantly higher rate of hrHPV positivity in women with AGC and concurrent squamous abnormalities compared to those with isolated AGC 4, 17, 19. Although our study did not identify a statistically significant difference in HPV positivity rates between AGC + Sq and AGC-Alone cases, the AGC + Sq group exhibited a higher hrHPV infection rate. Both groups predominantly harbored infections with one of the 12 other hrHPV types, consistent with data reported by Lin et al 17. Similarly, prior research has found no significant differences in the prevalence of HPV genotypes 16, 18, and 45 between patients with coexisting CIN3 and AIS and those with AIS alone. However, the overall hrHPV positivity rate was significantly higher in patients with both CIN3 and AIS 20. This suggests that while hrHPV positivity is more common in AGC + Sq cases, the specific genotype distribution may influence the clinical outcomes differently.

Our histologic results diverge slightly from those reported by Khor et al. 5 and Harbhajanka et al. 21. Khor et al. 5 documented that 51.7% of AGC with squamous abnormalities showed only squamous lesions upon histologic follow-up, while Harbhajanka et al. 21 reported 53.4%. Similarly, Lin et al. 17 found 41.5% of AGC + Sq cases exhibited only squamous lesions. In contrast, our study revealed that only 25.6% of cases showed solely squamous abnormalities, though this was still notably higher than the 14.6% in AGC-Alone cases. This discrepancy may be attributed to the smaller sample size in our study or incomplete follow-up data. Despite the rarity of concurrent squamous and glandular lesions, we observed an increased proportion of cases with both abnormalities in the AGC + Sq group compared to the AGC-Alone group (7.0% vs. 0.4%), aligning with Lin et al.'s results (8.0% vs. 0%) 17.

Furthermore, our data indicated that AGC + Sq cases had a significantly higher incidence of clinically significant histologic outcomes (Premalignancy and Malignancy) compared to AGC-Alone cases (30.2% vs. 15.0%). Notably, the proportion of cancer cases was higher in AGC + Sq patients (20.9%) compared to AGC-Alone patients (9.6%), highlighting a greater risk for severe histological outcomes. This may be attributed to a significantly higher proportion of AGC-FN cases within the AGC + Sq group. Among the cancer cases in the AGC + Sq group, 66.7% (6/9) were endometrial cancers, suggesting that clinicians should closely monitor endometrial lesions in these patients. These findings are consistent with those of Khor et al. 5, Pradhan et al. 18, and Harbhajanka et al. 21, who also reported significant rates of clinically significant histologic results in AGC + Sq patients.

Emerging evidence from recent studies suggests that evaluating the risk of precancerous lesions or cancer should extend beyond relying solely on cervical cytology and histological biopsy results 22. It is important to incorporate individual factors such as age into the assessment. By including additional variables, a more accurate stratification of patient risk can be achieved, thereby optimizing clinical management and decision-making. This study represents the first attempt to stratify risk in women with AGC based on specific hrHPV genotyping, addressing a gap in previous research that focused solely on the association between hrHPV infection status and pathological outcomes.

Similar to our previous research, the detection rates of clinically significant lesions were higher in HPV-16+ and HPV-18+ groups among AGC-Alone women and all AGC women 11. This is in contrast with the findings of Harbhajanka et al. 21, who found that HPV-positive AGC + Sq patients had significantly higher rates of glandular and squamous abnormalities compared to HPV-negative patients. Jang et al. 19 demonstrated that HPV positivity was significantly associated with the presence of cervical squamous or glandular lesions, while endometrial lesions were unrelated to HPV infection. Furthermore, Lin et al. 17 also observed a higher incidence of HSIL, AIS, or cancer among HPV-positive cases in the AGC cohort, particularly in the AGC + Sq group. In contrast, hrHPV infection status did not appear to affect histologic outcomes in AGC-Alone cases. These discrepancies may be due to differences in sample sizes, population demographics, or methodological approaches across studies.

Our previous study demonstrated a significant increase in the prevalence of glandular abnormalities among AGC women with advancing age 11. Similarly, Harbhajanka et al. noted an increase in glandular abnormalities with age and a decrease in squamous abnormalities 21. Our data indicate that increasing age significantly elevates the proportion of high-grade glandular lesions in AGC-Alone cases or across all AGC cases, but has no notable effect on AGC + Sq cases. Subsequent analysis, stratified by different age brackets with a cutoff at 50 years, revealed a significantly higher incidence of endometrial carcinoma in older AGC + Sq women compared to their younger counterparts, with the majority of these carcinomas being endometrial. While previous studies have explored the impact of AGC categories on histological outcomes, this study represents the first attempt to stratify by AGC categories 17, 19. We found that the detection rate of AIS+/AEH+ and AC was significantly higher in the AEM and AGC-FN subgroups among AGC + Sq women compared to other groups.

Our data further suggest that HPV testing may have limited utility as a primary screening tool for women with concurrent AGC and squamous lesions, as it may fail to detect significant precancerous and neoplastic changes. Additionally, both AGC subtypes and patient age substantially influence histologic follow-up outcomes. Therefore, regular monitoring is essential for AGC + Sq patients, particularly those in the AEM or AGC-FN subgroups aged over 50 (especially over 55), with subsequent hysteroscopic biopsy and appropriate intervention recommended. Furthermore, several studies have reported that patients with concurrent glandular and squamous lesions tend to have a more favorable prognosis, as evidenced by significantly lower recurrence rates compared to those with isolated glandular lesions 20, 23. Thus, accurately identifying these patients during routine cytologic evaluation is crucial for optimizing clinical management.

In conclusion, women presenting with AGC and concurrent squamous abnormalities exhibit a significantly higher proportion of squamous lesions upon histologic follow-up, as well as an increased risk of precursor lesions and malignancy compared to those with AGC alone. While hrHPV testing and patient age provide valuable information for assessing AGC, relying solely on hrHPV results for triaging AGC + Sq cases is inadequate. Specifically, AGC + Sq patients over the age of 50 should be closely monitored for the risk of glandular malignancies. A comprehensive approach that integrates cytologic evaluation, hrHPV status, and patient age may enhance risk stratification and inform more effective clinical management.

## Supplementary Material

Supplementary tables.

## Figures and Tables

**Table 1:** Demographic characteristics of patients with atypical glandular cells with and without concurrent squamous cell abnormalities.

Characteristic	AGC + Sq	AGC- Alone	*P*-vaule
Age, y	48.22 ± 12.79	45.25 ± 10.87	0.032
Age distribution, y			<0.0001
<25	0	7/974 (0.7%)	
25-39	15/54 (27.8%)	294/974 (30.2%)	
40-65	30/54 (55.6%)	614/974 (63.0%)	
>65	9/54 (16.7%)	59/974 (6.1%)	
AGC subtype, n/N (%)			<0.0001
AEM	9/54 (16.7%)	237/974 (24.3%)	
AEC	14/54 (25.9%)	275/974 (28.2%)	
AGC-NOS	9/54 (16.7%)	368/974 (37.8%)	
AGC-FN	22/54 (40.7%)	94/974 (9.7%)	
HPV status, n/N (%)			0.078
Positive	12/43 (27.9%)	131/756 (17.3%)	
Negative	31/43 (72.1%)	625/756 (82.7%)	
HPV type, n/N (%)			0.258
16+	3/12 (25.0%)	29/131 (22.1%)	
18+	1/12 (8.3%)	21/131 (16.0%)	
Other hrHPV+	7/12 (58.3%)	80/131 (61.1%)	
16+, 18+	1/12 (8.3%)	1/131 (0.8%)	
Histopathological Outcome			<0.0001
Negative/Benign lesion	30/43 (69.8%)	617/726 (85.0%)	
Premalignancy	4/43 (9.3%)	39/726 (5.4%)	
Malignancy	9/43 (20.9%)	70/726 (9.6%)	
Histopathological Outcome			0.001
Benign	19/43 (44.2%)	409/726 (56.3%)	
Gl A	10/43 (23.3%)	208/726 (28.7%)	
Sq A	11/43 (25.6%)	106/726 (14.6%)	
Gl A and Sq A	3/43 (7.0%)	3/726 (0.4%)	

AEC: atypical endocervical cells; AEM: atypical endometrial cells; AGC, atypical glandular cell; AGC + Sq, atypical glandular cells with concurrent squamous cell abnormalities; AGC-alone, atypical glandular cells without concurrent squamous cell abnormalities; AGC-NOS: atypical glandular cells, not otherwise specified; AGC-FN: atypical glandular cells, favor neoplastic; Gl A and Sq A, glandular abnormalities and squamous abnormalities; HPV, human papillomavirus.

**Table 2:** The prevalence of precancer and cancer among patients with atypical glandular cells with and without concurrent squamous cell abnormalities according to hrHPV types.

Group			Squamous lesions				Glandular lesions			
	hrHPV	Total	HSIL+, No. (%)	*p*	SCC, No. (%)	*p*	AIS+/AEH+, No. (%)	*p*	AC, No. (%)	*p*
AGC + Sq	negative	26	1 (3.9%)	0.276	0	NS	5 (19.2%)	0.629	4 (15.4%)	0.804
	16+	3	1 (33.3%)		0		1 (33.3%)		1 (33.3%)	
	18+	0	0		0		0		0	
	Other+	6	0		0		2 (33.3%)		1 (16.7%)	
	16+, 18+	1	0		0		0		0	
	Total	36	2 (5.6%)		0		8 (22.2%)		6 (16.7%)	
AGC-Alone	negative	514	3 (0.6%)	<0.0001	0	<0.0001	51 (9.9%)	<0.0001	38 (7.4%)	0.0043
	16+	25	8 (32.0%)		2 (8.0%)		7 (28.0%)		4 (16.0%)	
	18+	20	4 (20.0%)		2 (10.0%)		10 (50.0%)		3 (15.0%)	
	Other+	74	6 (8.1%)		1 (1.4%)		3 (4.1%)		3 (4.1%)	
	16+, 18+	1	0		0		1 (100%)		1 (100%)	
	Total	634	21 (3.3%)		5 (0.8%)		72 (11.4%)		53 (8.4%)	
AGC	negative	540	4 (0.7%)	<0.0001	0	<0.0001	56 (10.4%)	<0.0001	42 (7.8%)	0.0089
	16+	28	9 (32.1%)		2 (7.1%)		8 (28.6%)		5 (17.9%)	
	18+	20	4 (20.0%)		2 (10.0%)		10 (50.0%)		3 (15.0%)	
	Other+	80	6 (7.5%)		1 (1.3%)		5 (6.3%)		4 (5.0%)	
	16+, 18+	2	0		0		1 (50%)		1 (50%)	
	Total	670	23 (3.4%)		5 (0.8%)		80 (11.9%)		59 (8.8%)	

AC, adenocarcinoma; AEH, atypical endometrial hyperplasia; AGC, atypical glandular cell; AGC + Sq, atypical glandular cells with concurrent squamous cell abnormalities; AGC-alone, atypical glandular cells without concurrent squamous cell abnormalities; AIS, cervical adenocarcinoma in situ; hrHPV, high risk human papillomavirus; HSIL, high-grade squamous intraepithelial lesion; SCC, squamous cell carcinoma; NS, not significant.

**Table 3:** The prevalence of precancer and cancer among patients with atypical glandular cells with and without concurrent squamous cell abnormalities according to AGC categories.

Group			Squamous lesions				Glandular lesions			
	Categorie	Total	HSIL+, No. (%)	*p*	SCC, No. (%)	*p*	AIS+/AEH+, No. (%)	*p*	AC, No. (%)	*p*
AGC + Sq	AEM	8	0	1.000	0	NS	3 (37.5%)	0.011	3 (37.5%)	0.012
	AEC	13	1 (7.7%)		0		0		0	
	AGC-NOS	5	0		0		1 (20.0%)		0	
	AGC-FN	17	1 (5.9%)		0		8 (47.1%)		6 (35.3%)	
	Total	43	2 (4.7%)		0		12 (27.9%)		9 (20.9%)	
AGC-Alone	AEM	179	1 (0.6%)	0.002	0	0.002	18 (10.1%)	<0.0001	16 (8.9%)	<0.0001
	AEC	210	6 (2.9%)		1 (0.5%)		15 (7.1%)		4 (1.9%)	
	AGC-NOS	258	8 (3.1%)		1 (0.4%)		15 (5.8%)		12 (4.7%)	
	AGC-FN	79	6 (7.6%)		3 (3.8%)		41 (51.9%)		33 (41.8%)	
	Total	726	21 (2.9%)		5 (0.7%)		89 (12.3%)		65 (9.0%)	
AGC	AEM	187	1 (0.5%)	0.001	0	0.004	21 (11.2%)	<0.0001	19 (10.2%)	<0.0001
	AEC	223	7 (3.1%)		1 (0.5%)		15 (6.7%)		4 (1.8%)	
	AGC-NOS	263	8 (3.0%)		1 (0.4%)		16 (6.1%)		12 (4.6%)	
	AGC-FN	96	7 (7.3%)		3 (3.1%)		49 (51.0%)		39 (40.6%)	
	Total	769	23 (3.0%)		5 (0.7%)		101 (13.1%)		74 (9.6%)	

AC, adenocarcinoma.; AEC: atypical endocervical cells; AEM: atypical endometrial cells; AEH, atypical endometrial hyperplasia; AGC, atypical glandular cell; AGC + Sq, atypical glandular cells with concurrent squamous cell abnormalities; AGC-alone, atypical glandular cells without concurrent squamous cell abnormalities; AGC-NOS, atypical glandular cells, not otherwise specified; AGC-FN, atypical glandular cells, favor neoplastic; AIS, cervical adenocarcinoma in situ; hrHPV, high risk human papillomavirus; HSIL, high-grade squamous intraepithelial lesion; SCC, squamous cell carcinoma; NS, not significant.

**Table 4:** Age-stratified immediate histopathological correlation among women with AGC cytology with and without concurrent squamous cell abnormalities.

Group			Squamous lesions				Glandular lesions			
	Age, y	Total	HSIL+, No. (%)	*p*	SCC, No. (%)	*p*	AIS+/AEH+, No. (%)	*p*	AC, No. (%)	*p*
AGC + Sq	25-39	12	0	0.294	0	NS	4 (33.3%)	0.403	2 (16.7%)	0.191
	40-65	25	1 (4.0%)		0		5 (20.0%)		4 (16.0%)	
	>65	6	1 (16.7%)		0		3 (50.0%)		3 (50.0%)	
	Total	43	2 (4.7%)		0	12 (27.9%)	9 (20.9%)			
AGC-Alone	<25	4	0	1.000	0	0.910	1 (25.0%)	0.002	1 (25.0%)	<0.0001
	25-39	206	6 (2.9%)		1 (0.5%)		19 (9.2%)		7 (3.4%)	
	40-65	477	14 (2.9%)		4 (0.8%)		57 (12.0%)		46 (9.6%)	
	>65	39	1 (2.6%)		0		12 (30.8%)		11 (28.2%)	
	Total	726	21 (2.9%)		5 (0.7%)		89 (12.3%)		65 (9.0%)	
AGC	<25	4	0	0.791	0	0.909	1 (25.0%)	0.000444	1 (25.0%)	<0.0001
	25-39	218	6 (2.8%)		1 (0.5%)		23 (10.6%)		9 (4.1%)	
	40-65	502	15 (3.0%)		4 (0.8%)		62 (12.4%)		50 (10.0%)	
	>65	45	2 (4.4%)		0		15 (33.3%)		14 (31.1%)	
	Total	769	23 (3.0%)		5 (0.7%)	101 (13.1%)	74 (9.6%)			

AC, adenocarcinoma; AEH, atypical endometrial hyperplasia; AGC, atypical glandular cell; AGC + Sq, atypical glandular cells with concurrent squamous cell abnormalities; AGC-alone, atypical glandular cells without concurrent squamous cell abnormalities; AIS, cervical adenocarcinoma in situ; hrHPV, high risk human papillomavirus; HSIL, high-grade squamous intraepithelial lesion; SCC, squamous cell carcinoma; NS, not significant.

**Table 5:** Immediate risk of precancer and cancer between older and younger group with AGC cytology with and without concurrent squamous cell abnormalities.

Group			Squamous lesions				Glandular lesions			
	Age, y	Total	HSIL+, No. (%)	*p*	SCC, No. (%)	*p*	AIS+/AEH+, No. (%)	*p*	AC, No. (%)	*p*
AGC + Sq	40-year cutoff			0.604		NS		0.715		0.707
	≥40 years	31/43	2 (6.5%)		0		8 (25.8%)		7 (22.6%)	
	45-year cutoff			0.502		NS		0.804		0.381
	≥45 years	26/43	2 (7.7%)		0		8 (30.8%)		7 (26.9%)	
	50-year cutoff			0.133		NS		0.174		0.019
	≥50 years	18/43	2 (11.1%)		0		7 (38.9%)		7 (38.9%)	
	55-year cutoff			0.490		NS		0.277		0.046
	≥55 years	14/43	1 (7.1%)		0		6 (42.9%)		6 (42.9%)	
AGC-Alone	40-year cutoff			0.890		0.630		0.145		0.002
	≥40 years	516/726	15 (2.9%)		4 (0.8%)		69 (13.4%)		57 (11.1%)	
	45-year cutoff			0.825		0.885		0.000024		<0.0001
	≥45 years	361/726	9 (2.5%)		3 (0.8%)		63 (17.5%)		54 (15.0%)	
	50-year cutoff			0.957		0.553		0.00004		<0.0001
	≥50 years	223/726	6 (2.7%)		2 (0.9%)		44 (19.7%)		42 (18.8%)	
	55-year cutoff			1.000		0.539		0.000001		<0.0001
	≥55 years	122/726	3 (2.5%)		1 (0.8%)		31 (25.4%)		30 (24.6%)	
AGC	40-year cutoff			0.819		0.636		0.212		0.002
	≥40 years	547/769	17 (3.1%)		4 (0.7%)		77 (14.1%)		64 (11.7%)	
	45-year cutoff			0.960		0.904		0.000017		<0.0001
	≥45 years	387/769	11 (2.8%)		3 (0.8%)		71 (18.3%)		61 (15.8%)	
	50-year cutoff			0.498		0.566		0.000008		<0.0001
	≥50 years	241/769	8 (3.3%)		2 (0.8%)		51 (21.2%)		49 (20.3%)	
	55-year cutoff			0.968		0.553		<0.0001		<0.0001
	≥55 years	136/769	4 (2.9%)		1 (0.7%)		37 (27.2%)		36 (26.5%)	

AC, adenocarcinoma; AEH, atypical endometrial hyperplasia; AGC, atypical glandular cell; AGC + Sq, atypical glandular cells with concurrent squamous cell abnormalities; AGC-alone, atypical glandular cells without concurrent squamous cell abnormalities; AIS, adenocarcinoma in situ; hrHPV, high risk human papillomavirus; HSIL, high-grade squamous intraepithelial lesion; SCC, squamous cell carcinoma; NS, not significant.
