# Supplementary material for: HPV Genotype, AGC Categories, and Age-Stratified Immediate Prevalence of Precancers and Cancers in Women with Atypical Glandular Cells with or without Concurrent Squamous Abnormal Cytology
Source: J Cancer. 2025 Mar 31;16(7):2250–60. doi: 10.7150/jca.105805 (PMC12036100; doi:10.7150/jca.105805)
Supplement: Supplementary file 1 — Supplementary tables. [file jcav16p2250s1.pdf]

**Table S1. Demographic characteristics of patients with atypical glandular cells with concurrent squamous cell abnormalities.**			
**Characteristic**	**AGC with ASC-US/LSIL**	**AGC with ASC-H/HSIL**	***p*-Vaule**
Age, y	49.33 ± 12.89	43.90 ± 12.79	**<0.0001**
Age, y			0.640
25–39	11/43 (25.6%)	4/11 (36.4%)	
40–65	24/43 (55.8%)	6/11 (54.5%)	
>65	8/43 (18.6%)	1/11 (9.1%)	
AGC subtype, n/N (%)			0.617
AEM	8/43 (18.6%)	1/11 (9.1%)	
AEC	12/43 (27.9%)	2/11 (18.22%)	
AGC-NOS	6/43 (14.0%)	3/11 (27.3%)	
AGC-FN	17/43 (39.5%)	5/11 (45.5%)	
HPV status, n/N (%)			0.409
Positive	8/34 (23.5%)	4/9 (44.4%)	
Negative	26/34 (76.5%)	5/9 (55.6%)	
HPV type, n/N (%)			0.406
16+	3/8 (37.5%)	0	
18+	0	1/4 (25.0%)	
Other hrHPV+	4/8 (50.0%)	3/4 (75.0%)	
16+,18+	1/8 (12.5%)	0	
Histopathological Outcome			0.063
Negative/Benign lesion	26/37 (70.3%)	4/6 (66.7%)	
Premalignancy	2/37 (5.4%)	2/6 (33.3%)	
Malignancy	9/37 (24.3%)	0	
Histopathological Outcome			0.157
Benign	18/37 (48.7%)	1/6 (16.7%)	
Gl A	10/37 (27.0%)	1/6 (16.7%)	
Sq A	8/37 (21.6%)	4/6 (66.7%)	
Gl A and Sq A	1/37 (2.7%)	0	

AEC, atypical endocervical cells; AEM, atypical endometrial cells; AGC, atypical glandular cell; AGC-NOS, atypical glandular cells, not otherwise specified; AGC-FN, atypical glandular cells, favor neoplastic; ASC-US, atypical squamous cells of undetermined significance; ASC-H, atypical squamous cells, cannot exclude high-grade squamous intraepithelial lesion; Gl A and Sq A, glandular abnormalities and squamous abnormalities; HPV, human papillomavirus; HSIL, high-grade squamous intraepithelial lesion; LSIL, low-grade squamous intraepithelial lesion; NS, not significant.

Table S2. **Clinically significant histologic results in AGC patients with ASC-US/LSIL and ASC-H/HSIL according to HPV status and Age.**

Histological follow-up	AGC with ASC-US/LSIL			AGC with ASC-H/HSIL		
	**HPV-**	**HPV+**	***p*-Values**	**HPV-**	**HPV+**	***p*-Values**
Negative/benign	18 (78.3%)	5 (71.4%)	0.901	2 (66.7%)	1 (50.0%)	1.000
Premalignancy	1 (4.3%)	0		1 (33.3%)	1 (50.0%)	
Malignancy	4 (17.4%)	2 (28.6%)		0	0	
Total	23	7		3	2	
	**<40 years**	**≥40 years**	***p*-Values**	**<40 years**	**≥40 years**	***p*-Values**
Negative/benign	7 (70.0%)	19 (70.4%)	1.000	1 (50.0%)	3 (75.0%)	1.000
Premalignancy	1 (10.0%)	1 (3.7%)		1 (50.0%)	1 (25.0%)	
Malignancy	2 (20.0%)	7 (25.9%)		0	0	
Total	10	27		2	4	
	**<50 years**	**≥50 years**	***p*-Values**	**<50 years**	**≥50 years**	***p*-Values**
Negative/benign	18 (81.8%)	8 (53.3%)	**0.032**	2 (66.7%)	2 (66.7%)	1.000
Premalignancy	2 (0.9%)	0		1 (33.3%)	1 (33.3%)	
Malignancy	2 (0.9%)	7 (46.7%)		0	0	
Total	22	15		3	3	

AGC, atypical glandular cell; ASC-US, atypical squamous cells of undetermined significance; ASC-H, atypical squamous cells, cannot exclude high-grade squamous intraepithelial lesion; HPV, human papillomavirus; HSIL, high-grade squamous intraepithelial lesion; LSIL, low-grade squamous intraepithelial lesion; NS, not significant.

Table S3. The prevalence of precancer and cancer among patients with atypical glandular cells with and without concurrent squamous cell abnormalities according to hrHPV status.

Group			Squamous lesions				Glandular lesions			
	**hrHPV**	**Total**	**HSIL+, No. (%)**	***p***	**SCC, No. (%)**	***p***	**AIS+/AEH+, No. (%)**	***p***	**AC, No. (%)**	***p***
**AGC + Sq**	**Negative**	26	1 (3.9%)	0.483	0	NS	5 (19.2%)	0.849	4 (15.4%)	1.000
	**Positive**	10	1 (10.0%)		0		3 (30.0%)		2 (20.0%)	
	**Total**	36	2 (5.6%)		0		8 (22.2%)		6 (16.7%)	
**AGC-Alone**	**Negative**	514	3 (0.6%)	**<0.0001**	0	**0.000156**	51 (9.9%)	**0.002**	38 (7.4%)	0.252
	**Positive**	120	18 (15%)		5 (4.2%)		21 (17.5%)		11 (9.2%)	
	**Total**	756	21 (3.3%)		5 (0.8%)		72 (11.4%)		49 (6.5%)	
**AGC**	**Negative**	540	4 (0.7%)	**<0.0001**	0	**0.000173**	56 (10.4%)	**0.002**	42 (7.8%)	0.191
	**Positive**	130	19 (14.6%)		5 (3.9%)		24 (18.5%)		13 (10.0%)	
	**Total**	670	23 (3.4%)		5 (0.8%)		80 (11.9%)		55 (8.2%)	

AC, adenocarcinoma; AEH, complex atypical endometrial hyperplasia; AGC, atypical glandular cell; AGC + Sq, atypical glandular cells with concurrent squamous cell abnormalities; AGC-alone, atypical glandular cells without concurrent squamous cell abnormalities; AIS, cervical adenocarcinoma in situ; hrHPV, high risk human papillomavirus; HSIL, high-grade squamous intraepithelial lesion; SCC, squamous cell carcinoma; NS, not significant.
